# Usefulness of a commercial LAMP assay for detection of malaria infection, including *Plasmodium knowlesi* cases, in returning travelers in Spain

**DOI:** 10.1186/s13104-022-06037-9

**Published:** 2022-04-25

**Authors:** Alexandra Martín-Ramírez, Marta Lanza, Shamilah Hisam, Ana Perez-Ayala, José M. Rubio

**Affiliations:** 1 Malaria & Parasitic Emerging Diseases Laboratory, National Microbiology Center, Instituto de Salud Carlos III, Madrid, Spain; 2 Institute Medical Research, Parasitology Unit, Kuala Lumpur, Malaysia; 3 Department of Clinical Microbiology, Hospital Universitario, 12 de Octubre, Madrid, Spain

**Keywords:** Malaria, *Plasmodium*, Molecular diagnosis, *Plasmodium knowlesi*, LAMP, *Illumigene*^®^ Malaria, *Alethia*^®^ Malaria, PCR

## Abstract

**Objective:**

Main malaria diagnosis is based on microscopic examination combined with rapid diagnostic tests. Both methods have low sensitivity and specificity. Loop-mediated isothermal amplification techniques have shown a sensitivity similar to PCR but with lower times of performance. This study aimed to assess a commercial LAMP for the diagnosis of malaria (Alethia^®^ Malaria) against the Nested-Multiplex-Malaria PCR, including the analytical sensitivity and the operational characteristics.

**Results:**

One hundred five samples out of 114 rendered valid results, obtaining 85 positive samples and 18 negative samples with an agreement of 98% compared to the reference method with a sensitivity, specificity and kappa coefficient of 98.84%, 94.74% and 0.94 respectively, with only two discrepant samples. The turnaround time was estimated in 1 h and 30 min, with a cost of 32.67€ per determination. The results showed several advantages of the *Alethia*^®^ Malaria, as it was easy to perform, minimal training requirement and 40 min run. Moreover, it includes an internal control to avoid false negatives. However, it also showed some limitations such as the need for a specific amplification and detection device, the detection of only *Plasmodium* spp. and a very high price.

## Introduction

According to the last World Malaria Report, in 2020 an estimated 241 million cases of malaria occurred worldwide [1]. On the way towards elimination, one of the major issues to be addressed is the development of highly sensitive, reliable and easy-to-perform methods for the point-of-care diagnosis of malaria [1, 2].

In non-endemic countries, the majority of cases are imported [3] and locally acquired infections are rare events [4]. In Spain, malaria was declared officially eliminated in 1964 [5]. Since then, all reported cases have been imported, except for a few cases of locally acquired malaria [6]. Malaria diagnosis in non-endemic countries is challenging, due to the lack of experience and low levels of parasite densities, which lead to the chance of false negative results [7].

Conventional methods for malaria diagnosis are microscopic examination of Giemsa-stained thick and thin blood smears, often combined with rapid diagnostic tests (RDTs) [8]. Microscopy of thick blood smear remains the gold standard diagnosis of malaria according to the WHO. However, it is a labour intense method whose sensitivity strongly depends on the experience of microscopists [9]. Maintaining well experienced personal in malaria microscopy in non-endemic countries, where relatively few cases of malaria are seen, is difficult, which leads to false negative results and a heavy reliance on RDTs. On the other hand, RDTs are rapid, easy to use and sensitive for *P. falciparum;* nevertheless they lack sensitivity at low parasite density, fail to detect non-falciparum malaria and do not allow quantification of parasites [2, 8, 9].

Nucleic acid-based detection of malaria parasites (NAT) is now routine in reference laboratories of several non-malaria endemic countries, mostly based on polymerase chain reaction (PCR) [8]. However, novel techniques based on isothermal methods have been designed [10–12]. LAMP method amplifies DNA with high specificity, efficiency and rapidity under isothermal conditions [13, 14]. There are several LAMP commercial kits developed to detect malaria parasites [12, 15, 16]. Many recent reports, highlight the possible use of these commercial assays in clinical laboratories in non-endemic countries for malaria diagnosis, considering its high negative predictive value [7, 9, 17]. One of these commercial LAMP kits is Alethia^®^ Malaria LAMP assay (Meridian Bioscience), previously called *illumigene*^®^ Malaria, which is a qualitative in vitro diagnostic LAMP test for detection of *Plasmodium* spp. that has been tested in endemic [15] and in non-endemic malaria countries [2, 16, 18, 19]. However, most of the published studies assessing *Plasmodium* spp. samples using Alethia^®^ Malaria LAMP assay included none or very few samples of *Plasmodium knowlesi* parasites [7, 19, 20].

This study aimed to assess the diagnostic performance of the Alethia^®^ Malaria LAMP assay for the five species of *Plasmodium* that infect humans commonly, including *P. knowlesi,* compared to the Nested-Multiplex-Malaria PCR (NM-PCR). This evaluation includes the performance of the tests, the analytical sensitivity and the operational characteristics.

## Main text

### Material and methods

#### Clinical samples and study design

Samples used belonged to a project approved by the Medical and Health Research Ethics Committee (CEIC) of the Hospital Universitario 12 de Octubre (No. CEtm: 18/021). *Plasmodium knowlesi* samples were collected in Malaysia under a project approved by the Medical Research Sub-Committee of the Malaysian Ministry of Health (NMRR-13-1064-18,189). One hundred fourteen blood samples, 93 malaria positive (65 *P. falciparum,* 6 *P. vivax,* 9 *P. ovale,* 4 *P. malariae* and 9 *P. knowlesi*) and 21 negative samples were tested. Samples were tested retrospectively, and they were refrigerated at 4 ºC until the moment of performing the experiments.

#### Alethia^®^ Malaria LAMP assay

Alethia^®^ Malaria LAMP kit includes the material to perform the DNA extraction, using a simple filtration method, and the LAMP reaction. The assay was performed according to the manufacturer’s instructions using the Illumipro-10^™^ (Meridian Bioscience) for the amplification and reading of the results, obtaining a positive, negative or invalid result in 40 min. The LAMP reaction consists of two tubes with all the lyophilized components, one for the amplification of *Plasmodium* spp. and the second as internal reaction control.

#### Nested-Multiplex-Malaria PCR (NM-PCR)

DNA purification from 200 µl of whole EDTA blood was performed using the QIAamp DNA mini blood kit (QIAGEN^®^, Inc.) according to the manufacturer’s instructions.

NM-PCR was performed using gel form tubes (BioMalar Kit, Biotools) according to the manufacturer and original authors’ recommendations [21, 22]. The method involves two multiplex PCR amplifications that target the small subunit rDNA gene of *Plasmodium*. The first reaction amplifies *Plasmodium* spp. and an internal amplification control and the second reaction enables the identification of the infecting species by the corresponding size of the amplified fragments in the agarose gel electrophoresis [22].

The parameters used for the amplification were an initial denaturation at 94 ºC for 7 min, followed by 40 cycles at 94 ºC for 20 s, 58 ºC for 20 s, and 72 ºC for 30 s. The last cycle was followed by an extension time at 72 ºC for 10 min. Conditions for the second PCR reaction were an initial denaturation at 94 ºC for 5 min, followed by 35 cycles at 94 ºC for 15 s, 53 ºC for 15 s, and 72 ºC for 20 s, finishing with an extension phase at 72 ºC for 10 min.

#### Limit of detection of Alethia^®^ Malaria assay (LoD)

LoD was determined, by duplicate, using a *P. falciparum* positive blood sample belonging to a patient with a parasitaemia of 7500 parasites/µl determined by microscopy. Briefly, the sample was serially tenfold diluted with blood from a Spanish patient negative for malaria and no story of travelling to endemic malaria areas. DNA was extracted from each dilution by simple filtration method included in the kit and then tested by Alethia^®^ Malaria assay twice. LoD was considered as the lowest parasite concentration in which both duplicates were positive.

#### Operational characteristics

Estimated costs for the Alethia^®^ Malaria assay and the NM-PCR method were referred to as the cost for reagents for one determination without including costs associated with labor or derived from duplicates or controls. Turnaround time was estimated as the time required from the initial moment the sample begins to be processed until obtaining the results, considering the possibility of more than one sample are included in the run; while hands-on work time was the time required for the staff to perform the assay.

#### Statistics

Sensitivity (S), specificity (E), positive (PPV) and negative predictive value (NPV) and Kappa coefficient (k) were analyzed with 95% confidence intervals using EPI Dat (3.1) software package [23].

### Results

One hundred five samples out of 114 rendered valid results while nine samples gave invalid results when Alethia^®^ Malaria assay was used, obtaining 85 positive samples and 18 negative samples with an agreement of 98% compared to the reference method (Table 1). Only two samples (1.9%) provided discrepant results, a *P. malariae*-infected sample characterized by the NM-PCR, which was negative by the LAMP method, and a negative sample which rendered a positive result by the LAMP (Table 1).

**Table 1:** Comparison of the *Alethia*^®^ Malaria kit and NM-PCR results

Alethia^*®*^ Malaria	NM-PCR						
	*P. falciparum*	*P. ovale*	*P. vivax*	*P. malariae*	*P. knowlesi*	Negative	Total
Positive	59	9	6	2	9	1	86
Negative	0	0	0	1	0	18	19
Invalide	6	0	0	1	0	2	9
Total	65	9	6	4	9	21	114

The values of sensitivity and specificity were 98.8% and 94.7% respectively and the Kappa coefficient was 0.94 (Table 2).

**Table 2:** Sensitivity, specificity, predictive values and kappa coefficient of *Alethia*^*®*^ Malaria assay compared to NM-PCR

	Value (%)*	95% CI
Sensitivity	98.84	95.99–100
Specificity	94.74	82.06–100
Positive predictive value	98.84	95.99–100
Negative predictive value	94.74	82.06–100
Kappa coefficient	0.94	0.85–1

*CI* Confidence interval

*Results of sensitivity, specificity, positive and negative predictive values and kappa coefficient were calculated over 105 samples, excluding invalid samples

The limit of detection of the Alethia^*®*^ Malaria assay was 0.075 parasites/µl.

The turnaround time for the Alethia^*®*^ Malaria assay was estimated in 1 h 30 min (30 min for DNA purification, 10 min for tubes preparation, 40 min for amplification and 10 min for results reading), while for NM-PCR, it was estimated in 6 h 15 min (1 h for the management of the samples and DNA purification, 15 min for the first PCR setup, with the tubes ready to use, 2 h to run the first PCR, 15 min for the second PCR setup, 2 h to run the second PCR, 30 min for the automated electrophoresis, and 15 min for the analysis of results).

The cost for the commercial assay was calculated as 32.67€, higher than for the “in-house” assay of 5€, although in other countries or institutions, the costs of kits may be lower (Table 3).

**Table 3:** Time and costs estimated for *Alethia*^*®*^ Malaria and NM-PCR assays

Technique	Turnaround time	Hands-on work	Reagent cost per test
*Alethia*^*®*^ Malaria assay	1 h 30 min	50 min	32.67€
NM-PCR	6 h 15 min	2 h	5.00€

### Discussion

There is a growing request for faster and more sensitive diagnostic methods for malaria. Conventional methods, such as microscopy and RDTs, lack sensitivity, while PCR-based methods, although very sensitive [8], require highly trained personnel and expensive equipment. In contrast, LAMP-based methods have proven to have a similar or higher sensitivity than PCR methods but with the need for less training and resources [19, 24]. Alethia^*®*^ Malaria kit is a qualitative commercial assay able to detect *Plasmodium* spp. based on LAMP technology [25]. Furthermore, the kit includes the components to isolate DNA from whole blood by centrifuge-free methods [15]. In our study, the comparison between LAMP and NM-PCR assays, with just two discrepant samples (1.9%), showed a very good correlation corroborated with the high values of sensitivity, specificity and predictive values, as well as the kappa value (Table 2). Regarding the two discrepant results, it is very difficult to assess the accuracy of the LAMP method because any meaningful evaluation must be involved in comparison with other methods of diagnosis, in this occasion the NM-PCR, which might themselves be wrong. The false-positive result could be due to DNA contamination during sample processing [26, 27] or even to be a true-positive [12]. LAMP contamination has been thoroughly mentioned in the literature [2, 9, 12, 28], mainly due to the high sensitivity of this technique. However, in this commercial LAMP assay tubes are just opened at the beginning of the process, contrary to nested-PCR method that exhibits a higher risk of contamination with the opening of the tubes between first and second PCR steps. In addition, in each run, a free-DNA sample was included, in the DNA extraction and in the amplification, and it did not produce amplification by the LAMP method, so possibly the second option of being a true positive result was more feasible. The false-negative result in the sample infected with *P. malariae* may be due to a low parasitaemia of the original sample or that the LAMP assay exhibits deficiencies for the detection of this species. In this study, only four *P. malariae*-infected samples were analyzed, of which only two (50%) were correctly characterized, meanwhile another was considered negative and the last gave an invalid result. Expanding the number of samples infected with *P. malariae* would be necessary to find the correct answer.

Similar good results in specificity and sensitivity have been shown in more studies in non-endemic countries [2, 16, 18, 20]. In a study performed in France [2], they obtained 100% of sensitivity and 98.13% specificity using real-time PCR as the reference method. Studies performed in North America obtained excellent sensitivity compared to microscopy [16] and PCR [19]. Moreover, the Alethia^*®*^ Malaria kit has also been evaluated in Senegal obtaining high sensitivity (97.2%) and a good specificity [15]. However, neither of these studies tested any *P. knowlesi* specimens. In our study, the nine *P. knowlesi*-infected samples were detected, without any invalid or false-negative results, confirming the ability of the Alethia^*®*^ Malaria LAMP to detect the main five *Plasmodium* human species.

Nine samples out of 114 provided invalid results. Several authors have described a similar situation where it was not possible to obtain a valid result with this LAMP assay [2, 19]. According to the manufacturer’s instructions, some possible reasons for the invalid results may be inhibitory specimens, improper sample preparation, reagent failure, instrument failure, dirty device or improperly seated. In our study, the availability of test devices prevented the retest of these samples with an invalid result, however they were obtained at the beginning of the study but not at the end, suggesting that the technologist’s level of training played a factor in the results probably due to improper mixing of the blood samples and buffers.

The Alethia^®^ Malaria kit showed a very good LoD value for *P. falciparum*, 0.075 parasites/µl, similar to reported for several malaria Nested PCRs [21, 29]. Other reports obtained different LoD depending, possibly, on the origin of the samples used; 2 parasites/µl [14] and 0.5 parasites/µl [18] with conditions similar to ours, or 0.1 parasites/µl using serial dilutions of *P. falciparum* cultures [2].

Several malaria LAMP assays have been described previously [10–13], most of them “homemade” assays with reproducibility problems and with difficult-to-pass quality assessment controls. Conversely, commercial LAMP kits for malaria detection present the reagents in lyophilized form to enhance stability under ambient conditions, facilitating the use and decreasing the need for high training. Furthermore, the Alethia^®^ Malaria kit incorporates the components to purify DNA from whole blood and an internal reaction control to discriminate false negatives from inhibition reactions. Unfortunately, contrary to the best feature of LAMP technology, the reaction must be run on a specific device. Despite this, given our results, with excellent sensitivity and specificity and with a reduced time of diagnosis, Alethia^®^ Malaria assay could be used as a good screening diagnosis method for malaria, as other authors have pointed out previously [14, 19, 20].

### Conclusions

In this study, we demonstrated the utility of the Alethia^®^ Malaria for detection of the five most common *Plasmodium* human species, including *P. knowlesi* which was detected in 100% of samples assessed. The analytical sensitivity obtained was very good and our evaluation showed several advantages of the assay, as it was easy to perform, minimal training was needed, DNA purification was simple, fast and included in the kit, and the amplification run was completed in 40 min. Moreover, it includes an internal control to avoid false negatives. However, it also showed some limitations such as the need for a special amplification and detection device, the detection of only *Plasmodium* spp. with no information about species or the level of parasitaemia and the higher price of each test compared to non-commercial assays.

## Limitations

Our study presented some limitations, as the retrospective analysis of the samples and the low number of samples evaluated, especially for non-falciparum species. In addition, mixed infections were not included in the assessment and we did not undertake a reproducibility analysis.

## Data Availability

The databases used and/or analyzed during the current study are available from the corresponding author on reasonable request.

## Abbreviations

EDTA: Ethylenediaminetetraacetic acid
LAMP: Loop-mediated isothermal amplification
LoD: Limit of detection
PCR: Polymerase chain reaction
NM-PCR: Nested-multiplex-malaria PCR
RDT: Rapid diagnostic test

## References

[1] WHO. World malaria report 2021. 2021.

[2] Ponce C, Kaczorowski F, Perpoint T, Miailhes P, Sigal A, Javouhey E (2017). Diagnostic accuracy of loop-mediated isothermal amplification (LAMP) for screening patients with imported malaria in a non-endemic setting. Parasite.

[3] Askling HH, Nilsson J, Tegnell A, Janzon R, Ekdahl K (2005). Malaria risk in travelers. Emerg Infect Dis.

[4] European Centre for Disease Prevention and Control, Stockholm. Hospital-acquired malaria infections in the European Union. 2018.

[5] Pletsch D (1965). Informe sobre una misión efectuada en España en septiembre-noviembre de 1963 destinada a la certificación de la erradicación del paludismo. Rev San Hig Pública.

[6] Velasco E, Gomez-Barroso D, Varela C, Diaz O, Cano R (2017). Non-imported malaria in non-endemic countries: a review of cases in Spain. Malar J.

[7] Burdino E, Calleri G, Ghisetti V (2019). Added value of loop-mediated isothermal amplification technology (LAMP) in real life for the diagnosis of malaria in travellers. J Travel Med.

[8] Chiodini PL (2014). Malaria diagnostics: now and the future. Parasitology.

[9] Antinori S, Ridolfo AL, Grande R, Galimberti L, Casalini G, Giacomelli A (2021). Loop-mediated isothermal amplification (LAMP) assay for the diagnosis of imported malaria: a narrative review. Infez Med.

[10] Port JR, Nguetse C, Adukpo S, Velavan TP (2014). A reliable and rapid method for molecular detection of malarial parasites using microwave irradiation and loop mediated isothermal amplification. Malar J.

[11] Patel JC, Lucchi NW, Srivastava P, Lin JT, Sug-aram R, Aruncharus S (2014). Field evaluation of a real-time fluorescence loop-mediated isothermal amplification assay, RealAmp, for the diagnosis of malaria in Thailand and India. J Infect Dis.

[12] Cuadros J, Martin Ramírez A, González IJ, Ding XC, Perez Tanoira R, Rojo-Marcos G (2017). LAMP kit for diagnosis of non-falciparum malaria in Plasmodium ovale infected patients. Malar J.

[13] Notomi T, Okayami H, Masubuchi H, Yonekawa T, Watanabe K, Amino N (2000). Loop-mediated isothermal amplification of DNA. Nucleic Acids Res.

[14] Morris U, Aydin-Schmidt B (2021). Performance and application of commercially available loop-mediated isothermal amplification (LAMP) kits in malaria endemic and non-endemic settings. Diagnostics.

[15] Lucchi NW, Gaye M, Diallo MA, Goldman IF, Ljolje D, Deme AB (2016). Evaluation of the illumigene malaria LAMP: a robust molecular diagnostic tool for malaria parasites. Sci Rep.

[16] Rypien C, Chow B, Chan WW, Church DL, Pillai DR (2017). Detection of plasmodium infection by the illumigene malaria assay compared to reference microscopy and real-time PCR. J Clin Microbiol.

[17] Charpentier E, Benichou E, Pagès A, Chauvin P, Fillaux J, Valentin A (2020). Performance evaluation of different strategies based on microscopy techniques, rapid diagnostic test and molecular loop-mediated isothermal amplification assay for the diagnosis of imported malaria. Clin Microbiol Infect.

[18] De Koninck A-S, Cnops L, Hofmans M, Jacobs J, Van den Bossche D, Philippé J (2017). Diagnostic performance of the loop-mediated isothermal amplification (LAMP) based illumigene^®^ malaria assay in a non-endemic region. Malar J.

[19] Ljolje D, Abdallah R, Lucchi NW (2021). Detection of malaria parasites in samples from returning US travelers using the Alethia^®^ malaria plus LAMP assay. BMC Res Notes.

[20] Frickmann H, Hinz R, Rojak S, Bonow I, Ruben S, Wegner C (2018). Evaluation of automated loop-mediated amplification (LAMP) for routine malaria detection in blood samples of German travelers–a cross-sectional study. Travel Med Infect Dis.

[21] Rubio JM, Post RJ, van Leeuwen WD, Henry M-C, Lindergard G, Hommel M (2002). Alternative polymerase chain reaction method to identify Plasmodium species in human blood samples: the semi-nested multiplex malaria PCR (SnM-PCR). Trans R Soc Trop Med Hyg.

[22] Miguel-Oteo M, Jiram AI, Ta-Tang TH, Lanza M, Hisam S, Rubio JM (2017). Nested multiplex PCR for identification and detection of human Plasmodium species including *Plasmodium knowlesi*. Asian Pac J Trop Med.

[23] Santiago Perez MI, Hervada Vidal X, Naveira Barbeito G, Silva LC, Fariñas H, Vázquez E (2010). The Epidat program. Rev Panam Salud Publica.

[24] Barazorda KA, Salas CJ, Bishop DK, Lucchi N, Valdivia HO (2020). Comparison of real time and malachite-green based loop-mediated isothermal amplification assays for the detection of *Plasmodium vivax* and *P. falciparum*. PLoS ONE..

[25] Meridiam Illumigene Marketing Collateral. Illumigene^®^ Malaria Package Insert, SN11102. REV. 01/16. 2017.

[26] Lau Y-L, Lai M-Y, Fong M-Y, Jelip J, Mahmud R (2016). Loop-mediated isothermal amplification assay for identification of five human *Plasmodium* species in Malaysia. Am J Trop Med Hyg.

[27] Morris U, Khamis M, Aydin-Schmidt B, Abass AK, Msellem MI, Nassor MH (2015). Field deployment of loop-mediated isothermal amplification for centralized mass-screening of asymptomatic malaria in Zanzibar: a pre-elimination setting. Malar J.

[28] Polley SD, Gonzalez IJ, Mohamed D, Daly R, Bowers K, Watson J (2013). Clinical evaluation of a loop-mediated amplification kit for diagnosis of imported malaria. J Infect Dis.

[29] Singh B, Bobogare A, Cox-Singh J, Snounou G, Abdullah MS, Rahman HA (1999). A genus-and species-specific nested polymerase chain reaction malaria detection assay for epidemiologic studies. Am J Trop Med Hyg.

